# Formation of a Nanorod-Assembled TiO_2_ Actinomorphic-Flower-like Microsphere Film via Ta Doping Using a Facile Solution Immersion Method for Humidity Sensing

**DOI:** 10.3390/nano13020256

**Published:** 2023-01-06

**Authors:** Musa Mohamed Zahidi, Mohamad Hafiz Mamat, A Shamsul Rahimi A Subki, Mohd Hanapiah Abdullah, Hamizura Hassan, Mohd Khairul Ahmad, Suriani Abu Bakar, Azmi Mohamed, Bunsho Ohtani

**Affiliations:** 1 NANO-ElecTronic Centre (NET), School of Electrical Engineering, College of Engineering, Universiti Teknologi MARA, Shah Alam 40450, Selangor, Malaysia; 2 Centre for Electrical Engineering Studies, Universiti Teknologi MARA Cawangan Pulau Pinang, Permatang Pauh 13500, Pulau Pinang, Malaysia; 3 Faculty of Electrical and Electronic Engineering Technology, Universiti Teknikal Malaysia Melaka, Hang Tuah Jaya, Durian Tunggal 76100, Melaka, Malaysia; 4 Centre for Chemical Engineering Studies, Universiti Teknologi MARA Cawangan Pulau Pinang, Permatang Pauh 13500, Pulau Pinang, Malaysia; 5 Microelectronic and Nanotechnology—Shamsuddin Research Centre, Faculty of Electrical and Electronic Engineering, Universiti Tun Hussein Onn Malaysia, Batu Pahat 86400, Johor, Malaysia; 6 Nanotechnology Research Centre, Faculty of Science and Mathematics, Universiti Pendidikan Sultan Idris, Tanjung Malim 35900, Perak, Malaysia; 7 Graduate School of Environmental Science, Hokkaido University, Sapporo 060-0810, Japan

**Keywords:** semiconductors, sol-gel preparation, TiO_2_ nanostructure, Ta doping, structural, sensors

## Abstract

This study fabricated tantalum (Ta)-doped titanium dioxide with a unique nanorod-assembled actinomorphic-flower-like microsphere structured film. The Ta-doped TiO_2_ actinomorphic-flower-like microsphere (TAFM) was fabricated via the solution immersion method in a Schott bottle with a home-made improvised clamp. The samples were characterised using FESEM, HRTEM, XRD, Raman, XPS, and Hall effect measurements for their structural and electrical properties. Compared to the undoped sample, the rutile-phased TAFM sample had finer nanorods with an average 42 nm diameter assembled to form microsphere-like structures. It also had higher oxygen vacancy sites, electron concentration, and mobility. In addition, a reversed double-beam photoacoustic spectroscopy measurement was performed for TAFM, revealing that the sample had a high electron trap density of up to 2.5 μmolg^−1^. The TAFM showed promising results when employed as the resistive-type sensing film for a humidity sensor, with the highest sensor response of 53,909% obtained at 3 at.% Ta doping. Adding rGO to 3 at.% TAFM further improved the sensor response to 232,152%.

## 1. Introduction

Titanium dioxide (TiO_2_) is a metal oxide compound used in various applications, including photocatalyst [1,2], solar cells [3,4], and sensors [5]. It has favourable attributes, including high chemical stability, non-toxicity, and good photocatalytic activity. A crucial advantage of TiO_2_ is its flexibility to be shaped into various types of structures [6]. Such structures with a high surface area are extremely useful in sensor applications as they can better detect analytes, particularly for sensors that rely on surface adsorption, such as gas and humidity. Chen et al. synthesised TiO_2_ nanorod structures as the mesoporous supporting layer in a perovskite solar cell [7]. Meanwhile, Jiang et al. produced TiO_2_ nanobelt structures using the hydrothermal method in an alkaline medium to be used in photoelectrochemical reactions to produce hydrogen gas [8]. 

Three-dimensional (3D) structures such as microspheres have been reported to provide a high surface area for zinc oxide (ZnO) [9], tin oxide (SnO_2_) [10], magnesium oxide (MgO) [11], and nickel oxide (NiO) [12,13]. However, reports on spherical TiO_2_ microstructures are limited. Lan et al. [14] produced mesoporous TiO_2_ microspheres through a pressure-driven hydrothermal method using an autoclave. However, their method required an amphiphilic triblock copolymer to produce the unique structure and the growth time was up to 24 h. Arjunkumar et al. [15] presented TiO_2_ microspheres, hydrothermally synthesised on a fluorine-doped tin oxide (FTO)-coated substrate. Their method also relied on an autoclave to generate pressure to form of 10 µm–sized TiO_2_ microspheres in 8 h of growth duration. Meanwhile, Ren et al. [16] produced a flower-like TiO_2_ microsphere using the hydrothermal method at a deposition time of 24 h. They utilised SiO_2_ as the template to form TiO_2_ hollow microspheres.

Based on the literature review, there is no report on the formation of nanorod-assembled Ta-doped TiO_2_ actinomorphic-flower-like microsphere (TAFM) using a simple solution immersion without an autoclave. Furthermore, the use of TAFM structures as sensing films for humidity-sensing applications has not been reported. This research discovered that this structure type could be formed by adding TaCl_5_. The advantage of using TaCl_5_ is that it can serve bifunctionally as both a Ta dopant source and a catalyst for the formation of microspheres. Pentavalent dopants, such as Ta, enhance the optical and electrical characteristics of TiO_2_ [17,18]. This method also eliminates the need for triblock copolymer or FTO-coated substrates and an autoclave, which have been used in other methods. Furthermore, this study’s method produced the microsphere in the shortest reported growth duration of only 4 h. This study enhanced the microstructure’s growth rate by replacing the autoclave with a Schott bottle.

This research included results from reversed double-beam photoacoustic spectroscopy (RDB-PAS) for TAFM, which has rarely been reported in the literature. Ohtani et al. introduced RDB-PAS, a novel method characterising a material’s electron trap density [19]. This technique excites electrons in the valence band of material to electron traps (ET) located in the band gap by wavelength-selective continuous light from the deeper side to the shallower side. Photoacoustic spectroscopy (PAS) then evaluates the increase in photo absorption using modulated LED light. The acquired spectrum was differentiated to reveal the sample’s energy-resolved distribution of ETs (ERDT) [20]. To the best of the authors’ knowledge, no literature has been published on the RDB-PAS analysis of TiO_2_ microspheres, and this work aimed to fulfil that gap. The properties of the TAFM, including structural, surface, electrical, and humidity responses, were investigated. This research also explored the humidity response of TAFM/reduced graphene oxide (rGO) nanocomposite film.

TiO_2_ and rGO were chosen for humidity sensor application in this study because these two materials possess excellent characteristics for water detection. TiO_2_ has a hydrophilic nature and chemical robustness, making it suitable for humidity detection. Meanwhile, rGO has been reported to improve the humidity detection of other metal oxide-based humidity sensors. In addition, carbon nanostructures can be prepared using abundant organic precursor materials, including waste materials [21,22]. TiO_2_ nanostructures with a high surface area exhibit excellent humidity-sensing ability. Jeong et al. [23] reported on a resistive humidity sensor fabricated using a flower-like TiO_2_ nanostructure. The 3D architecture was prepared via hydrothermal processing and deposited on a flexible polyimide substrate using the drop-casting method. They reported that the highest sensitivity obtained was attributed to the immense surface area of TiO_2_.

Researchers have also combined metal oxide with graphene to enhance humidity sensors’ performance further. Saqib et al. [24] used a composite of ZnO and graphene to produce a high-performance humidity sensor. The highly electrically conductive graphene provided more current flow between the electrodes. The addition of two-dimensional (2D) graphene also increased the structure’s total surface area, improving the sensor’s performance. Identical outcomes were also reported using SnO/rGO [25], TiO_2_/rGO [26], and MoS_2_/GO [27] composites. The current advancement in humidity sensor response capability has paved the way for its application in breath detection. It could allow non-invasive and real-time monitoring of human breathing conditions and respiratory-related diseases. Li et al. [28] demonstrated the feasibility of using polymer composite-based humidity sensors to detect a human breath. The device could distinguish a person’s slow, average, and fast breathing rates. In another work [29], a graphene-based device identified a person with rhinitis from the breath response pattern. This study synthesised a unique TAFM to fabricate a humidity sensor with the potential for application in detecting human breath. The TAFM was also combined with the rGO to improve the sensing performance further.

## 2. Materials and Methods

A 370 nm-thick seed layer of TiO_2_ was sputtered evenly on a 2.5 × 2.5 cm^2^ microscope glass slide using radio frequency (RF) magnetron sputtering (SNTEK) by bombarding plasma ions on a pure TiO_2_ target. The RF power was set at 200 W for 6 h under 20 sccm argon and 5 sccm oxygen. A solution containing 0.07 M of titanium butoxide (Ti(C_4_H_9_O)_4_, Purity: 97%, Sigma-Aldrich, USA) was prepared in a Schott bottle with a solvent-acid mixture of deionised (DI) water and hydrochloric acid. A small amount of tantalum pentachloride (TaCl_5_, Purity: 99.8%, Sigma-Aldrich, China) was added to the solution to achieve 1 at.%, 3 at.%, 5 at.%, 7 at.%, and 9 at.% Ta doping. An undoped solution was also prepared for comparison. After 1 h of rigorous stirring, a substrate was arranged into the immersion bottle with the TiO_2_-seed-layer-coated surface facing upwards. A unique, high-heat resistance cap was used to tightly seal the bottle to ensure pressure built up inside the bottle. The bottle was then clamped with a homemade clamp (Figure S1) made of two metal plates on the top and bottom of the bottle. Bolts and nuts secured both plates in place. The bottle was then put in a 150°C-hot oven. After 4 h of immersion, the sample was bathed with DI water and dried using nitrogen gas. The film underwent a post-annealing treatment at 500 °C for 1 h. The pristine and undoped sample was named UTD whereas the Ta-doped samples were named TAFM-1, TAFM-3, TAFM-5, TAFM-7, and TAFM-9 for 1 at.%, 3 at.%, 5 at.%, 7 at.%, and 9 at.% Ta doping, respectively. A nanocomposite of TAFM-3 and rGO was prepared according to a previously reported method [26] to assess the effect of rGO addition to Ta-doped TiO_2_. An aqueous rGO solution prepared at 0.03 mg/mL was dropped uniformly on TAFM-3 using a micropipette based on the drop-casting process. The excess solution was dried out on a hot-plate stirrer.

Field-emission scanning electron microscopy (FESEM, JEOL JSM6360LA, Japan), high-resolution transmission electron microscopy (HRTEM, FEI TECNAI G2 20 S-TWIN, Netherlands), and energy-dispersive X-ray spectroscopy (EDX, Oxford Instruments X-MAX80, England) examined the morphology, atomic structure, and elemental mappings of the prepared films, respectively. X-ray diffraction (XRD, Shimadzu XRD-6000, Japan, Cu-Kα radiation, wavelength of 0.154 nm) and Raman spectroscopy (Horiba Jobin Yvon-79 DU420A-OE-325, France, 514 nm Ar laser) determined the structural characteristics of the thin film. An X-ray photoelectron spectroscopy (XPS, Thermo Scientific Nexsa G2, USA) analysis of the samples was also performed to ascertain the samples’ chemical states. The survey and narrow scan were conducted using pass energies of 280 eV and 112 eV, respectively. RDB-PAS analysis was done using the method used by Nitta et al. [19]. Hall effect measurement (Nanomagnetics Instruments ezHEMS) was conducted on the samples for electrical characterisation. The current-voltage (I-V) measurements were performed using a Keithley 2400 sensor measurement system to gauge the performance of the materials as humidity sensors. The system was inside a humidity chamber (ESPEC-SH261, Japan) with thermally evaporating 60-nm thick silver (Ag) contact on the films. The Ag contacts were deposited with a physical mask for electrode patterning in the thermal evaporation process (ULVAC Thermal Evaporator, Japan). The transient humidity responses of the sensors were measured inside two humidity chambers with different humidity levels of 40% RH and 90% RH.

## 3. Results

**Figure 1:**
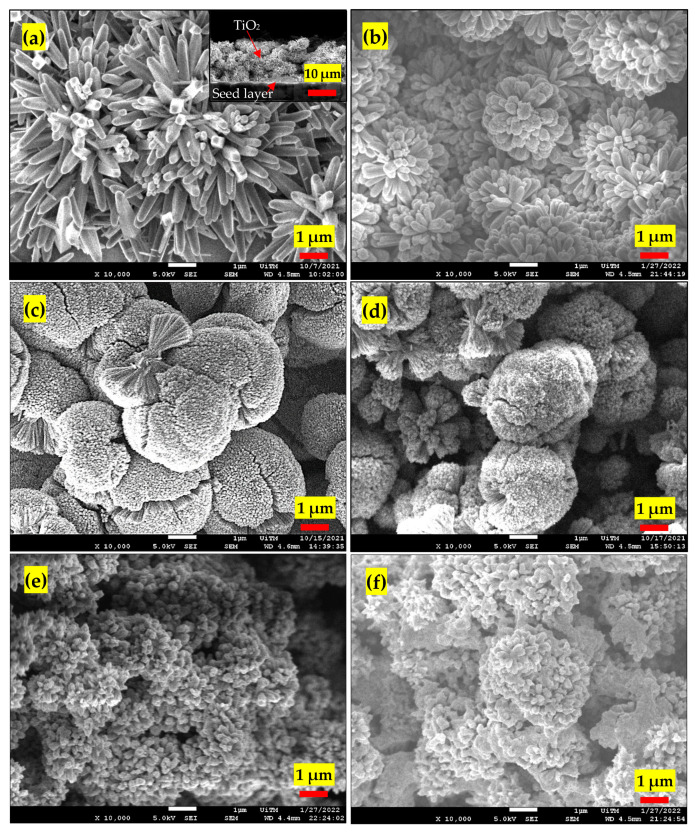
FESEM images of (**a**) UTD, inset cross-sectional view, (**b**) TAFM-1, (**c**) TAFM-3, (**d**) TAFM-5, (**e**) TAFM-7, and (**f**) TAFM-9 at 10,000× magnification.

The 10,000× magnified images of the prepared samples were taken by FESEM (Figure 1a–f). The pristine UTD exhibited a dendritic nanorod structure with an average nanorod diameter of 288 nm. The cross-sectional images of UTD (inset figure in Figure 1a) revealed that the 20-μm-thick film was uniformly grown on the glass substrate with a porous structure. As the Ta concentration increased, the nanorod formed an actinomorphic, flower-like-microsphere structure. A perfectly spherical shape was observed as the doping percentage reached 3 at.% and 5 at.%. The average microsphere diameters of TAFM-3 and TAFM-5 were approximately 4.1 and 3.9 µm, respectively. A further increase in doping concentration beyond that point resulted in TiO_2_ losing its microsphere structure and ending up with aggregated particles, as shown in Figure 1e,f.

Higher magnification images of UTD and TAFM-3 showed that the microsphere of TAFM-3 consisted of assembled nanorods with a diameter of around 42 nm (Figure S2a,b). The microspheres had a porous surface with a high surface area. Adding TaCl_5_ up to 5 at.% reduced the diameter of the nanorods and caused the nanorods to be aggregated to form actinomorphic flower-like structures.

**Figure 2:**
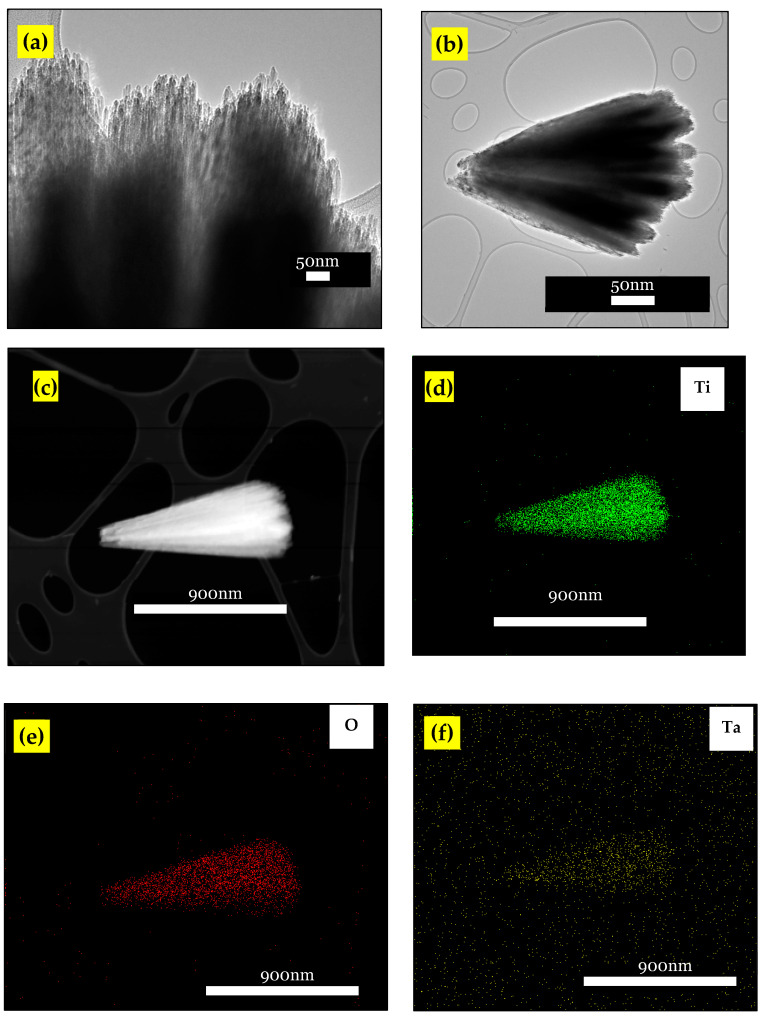
(**a**,**b**) High magnification HRTEM image of TAFM-3, and (**c**–**f**) EDX elemental mappings of TAFM-3.

The HRTIM image of the TAFM-3 (Figure 2a,b) revealed a TiO_2_ nanorod constructed from a 5 nm-diameter nanorod assembly. The EDX elemental mappings (Figure 2c–e) revealed that the TAFM sample had a uniform Ti and O distribution across the structure. The dopant, Ta, also appeared uniformly on the nanorod’s surface (Figure 2f).

**Figure 3:**
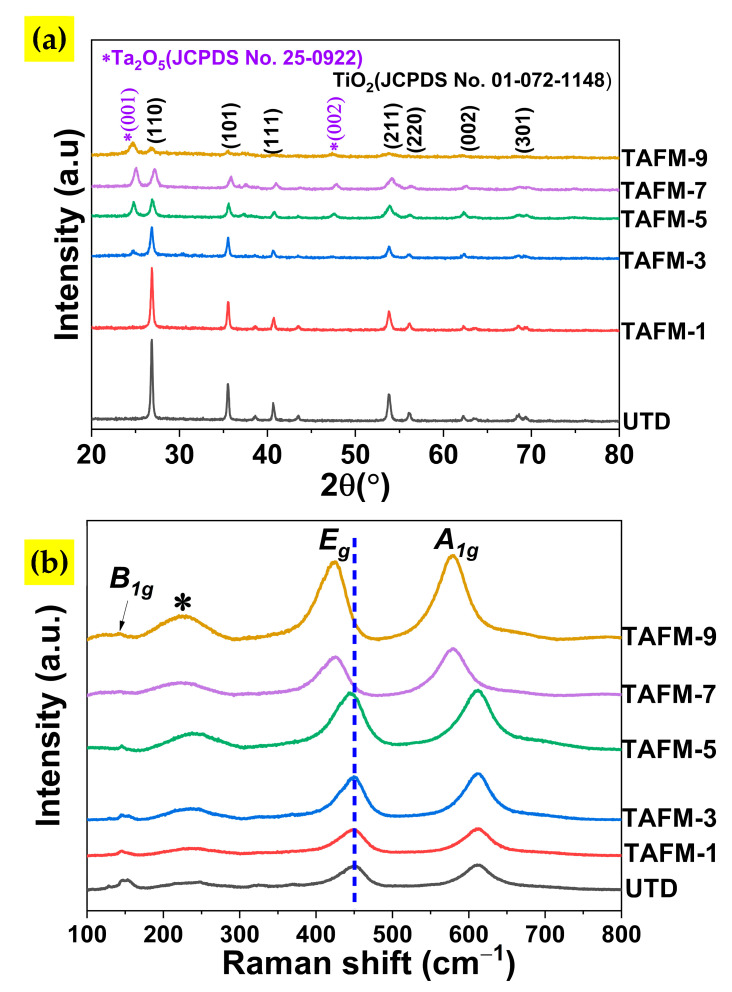
(**a**) XRD pattern of all samples and (**b**) Raman spectra of all samples.

The XRD result of the prepared samples (Figure 3a) specified that the synthesised films exhibited polycrystalline rutile tetragonal structured TiO_2_ with the appearance of several diffraction peaks associated with the (110), (101), (111), (211), (200), (002), and (301) planes. For samples with Ta doping concentrations of 3 at.% and above, peaks were observed at 2*θ* of 24.9° and 24.6° ascribed to Ta_2_O_5_. The interplanar spacing of *d_hkl_* and lattice constants, *a*, of both the undoped (UTD) and Ta-doped (TAFM) samples were determined by Equations (1) and (2) [30,31].

(1) $$\frac{1}{d_{hkl}{}^{2}}=\frac{h^{2}+k^{2}}{a^{2}}+\frac{l^{2}}{c^{2}}$$

(2) $$d_{hkl}=\frac{n\lambda }{2sin\theta }$$

where *a* is the lattice constant, *θ* is the diffraction angle, *n* is the diffraction order (usually *n* = 1), and *λ* is the X-ray wavelength (1.54 Å). Using the XRD peak of the (110) crystalline plane, the interplanar distance (*d_hkl_*) and lattice parameter (*a*) for UTD, TAFM-1, TAFM-3, TAFM-5, TAFM-7, and TAFM-9 were calculated to be 3.32 Å/4.691 Å, 3.31 Å/4.688 Å, 3.32 Å/4.692 Å, 3.31 Å/4.676 Å, 3.28 Å/4.638 Å, and 3.32 Å/4.695 Å, respectively, as shown in Table 1.

Scherrer’s equation was used to determine the crystallite size (*D*) (Equation (3)) [32]:

(3) $$D=\frac{0.94\lambda }{\beta cos\theta }$$

where *λ*, *β*, and *θ* represent the X-ray wavelength (1.54 Å), full width at half maximum (FWHM) of the (110) plane, and peak angle of the (110) plane, respectively. The crystallite size decreased as a higher concentration of Ta was added to TiO_2_. It is a common doping effect since foreign elements often disrupt the crystal growth of the parent material [33]. Meanwhile, the microstrain, *ε*, was calculated using Equation (4) [34]:

(4) $$\varepsilon =\frac{\beta }{4\tan\theta }$$

where *β* is the full width at half maxima (FWHM) of the (110) peak. The detailed analysis of XRD data revealed that TAFM samples had a higher microstrain than that of UTD. When examining the ionic radius, there was a slight mismatch in the sizes of Ti^4+^ (0.605 Å) and Ta^5+^ (0.640 Å), which could have promoted the defects. Other possible reasons are the electron density around Ta and the doping-induced oxygen vacancies, which could cause the rearrangement of nearby atoms and disrupt the crystal growth [35,36].

The raman spectroscopy results in Figure 3b showed that the vibration modes were observed at 143 cm^−1^, 235 cm^−1^, 447 cm^−1^, and 612 cm^−1^, which correspond to *B_1g_* and two-phonon bands (marked with*), *E_g_* and *A_1_*_g_, respectively. Conforming with the XRD results, this pattern indicated TiO_2_ with a rutile polymorph [37]. The *E_g_* peak was ascribed to the mode of the Ti–O vibration, whereas the vibration mode of the oxygen atoms along the c-axis was associated with the *A_1g_* peak. The broadening of the *E_g_* peak of the Ta-doped sample indicated the incorporation of Ta^5+^ into Ti^4+^ sites [38]. In addition, the *E_g_* shifted slightly to the lower wavenumber, indicating the increased oxygen vacancy [39,40].

**Figure 4:**
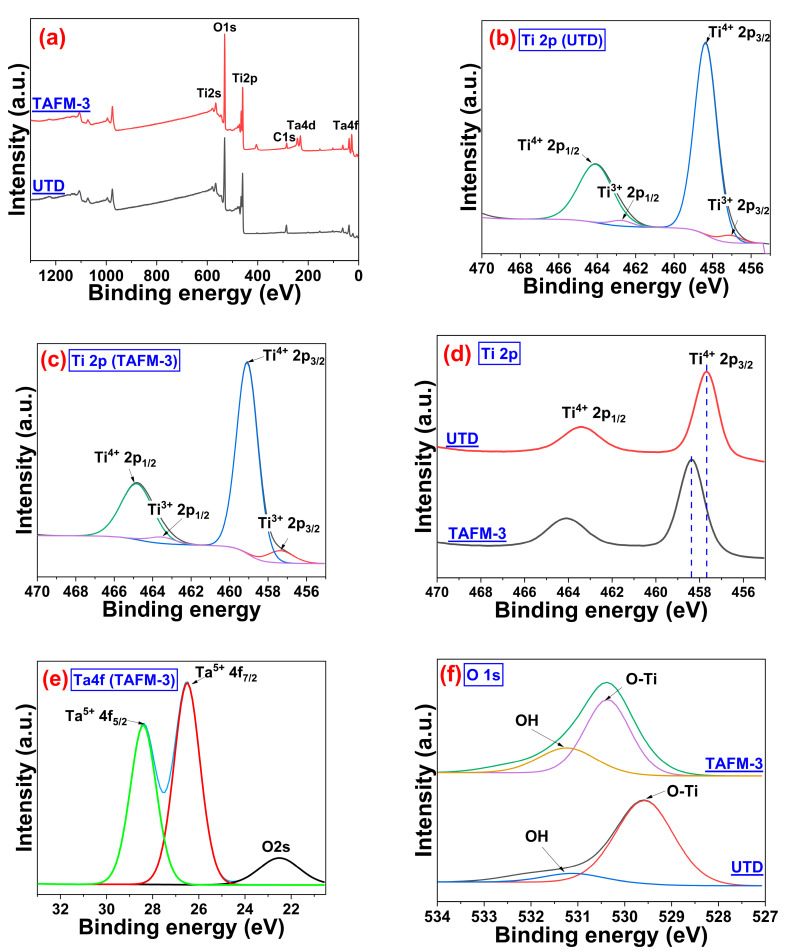
(**a**) XPS survey scan of UTD and TAFM-3, (**b**) Ti 2p scan of UTD, (**c**) Ti 2p scan of TAFM-3, (**d**) Ti 2p shift, (**e**) Ta4f scan of TAFM-3, and (**f**) O1s scan of UTD and TAFM-3.

The XPS analysis result of UTD and TAFM-3 is shown in Figure 4. The scan survey (Figure 4a) confirmed the presence of Ta in TAFM-3 by the appearance of the Ta 4f peak at a binding energy of 26 eV, which was absent in the undoped TiO_2_. The core-level scan of Ti for UTD (Figure 4b) revealed 2p_3/2_ and 2p_1/2_ peaks at binding energies of 458.35 eV and 464.08 eV, respectively, which corresponded to the Ti^4+^ oxidation state. Meanwhile, Ti^4+^ 2p_3/2_ and Ti^4+^ 2p_1/2_ peaks were located at 459.08 eV and 464.82 eV, respectively, for the TAFM-3 sample. Comparing the intensities of Ti^3+^ peaks between samples UTD and TAFM-3, it was deduced that the doping of Ta increased the Ti^3+^ oxidation state proportion in TiO_2_ [41]. It implied that incorporating Ta^5+^ ions increased the number of oxygen vacancies in TiO_2_ in the crystal structure [38]. The expanded plots of the Ti 2p_3/2_ peaks of UTD and TAFM-3 are shown in Figure 4d. The 2p_3/2_ peak of TAFM-3 was shifted to a slightly higher binding energy than UTD because of the intensification of electron-electron interactions and electron-hole interactions [17]. The Ta doping also contributed to Ti’s low valence level, which was associated with forming oxygen vacancies in the lattice [42]. In addition, the shifted peak position in the doped sample implied an effect of Ta doping on the Ti electronic state, whereby some of the Ti^4+^ ions were substituted for the Ta^5+^ ions in the TiO_2_ lattices, creating lattice distortion in the process [43,44].

The peak area of Ti^3+^ grew by 65% after Ta doping, whereas Ti^4+^ shrank by 13%. The expansion of the Ti^3+^ peak’s area suggested that a significant amount of Ti_2_O_3_ was produced or some mixed oxide structure with Ta was formed after Ta doping. The Ti^3+^ functioned as a Bronsted acid site, which attracted water molecules to form the OH–H_2_O complex [45]. The decreasing area of the Ti^4+^ peak indicated that there was less TiO_2_ in the sample and that the Ta^5+^ ions substituted the Ti^4+^ ions in the TiO_2_ lattice to form the Ti–O–Ta structure. In addition, the generation of oxygen vacancies in the TAFM-3′s surface layer also contributed to the decreasing area of the Ti^4+^ peak. The detailed scan of Ta (Figure 4e) showed two distinct peaks at binding energies of 26.5 eV and 28.4 eV, corresponding to Ta^5+^ 4f_7/2_ and Ta^5+^ 4f_5/2_, respectively.

The narrow scan of O 1s contained an O–Ti peak at around 529.57 eV and 530.37 eV for UTD and TAFM-3 (Figure 4f). Another lower-intensity peak appeared at 531.2 eV and was ascribed to the presence of the hydroxyl group (OH) [46,47]. This OH group was generated from dissociated water adsorption on oxygen vacancies [45]. The increase of the OH peak in the TAFM-3 sample compared to that in the UTD sample indicated an upsurge of oxygen vacancies on the TAFM-3′s surface and improved water affinity characteristics of the Ta-doped sample’s surface.

**Figure 5:**
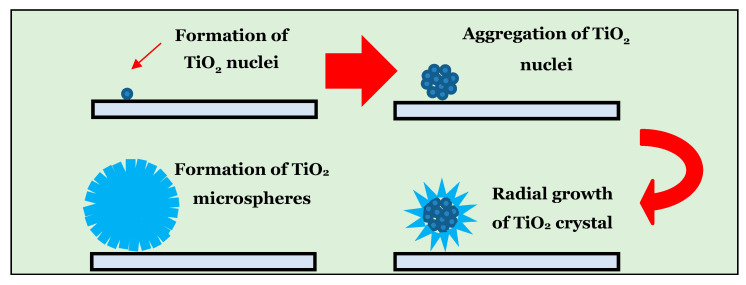
Growth mechanism of TiO_2_ microsphere.

The growth mechanism of TAFM’s microsphere (Figure 5) was initiated with TiO_2_ nuclei forming on the TiO_2_ seed layer. These nuclei aggregated to form sphere-shaped microstructures. Over time, nanoparticles at the surface of the microspheres dissolved before recrystallising to form nanorods that grew radially outward. This dissolution and recrystallisation process occurred through crystallographic fusion to obtain a more stable thermodynamic state [48]. Adding TaCl_5_ salt increased the ionic strength, which hindered the hydrolysis of TiO^2+^ ions in the solution. It resulted in a lower number of nuclei available for further growth of TiO_2_. Meanwhile, Cl^−^ ions were absorbed and inhibited the growth rate in the (110) plane while advancing the anisotropic growth in the (001) direction.

The results for RDB-PAS measurements (Figure S3) showed that as the continuous light wavelength was scanned from longer to shorter wavelengths, the photoacoustic intensity slowly increased and reached saturation around 350 nm (Figure S3a). Nitta et al. reported a similar result [19]. The RDB-PAS spectra (Figure S3b) were related to the integrated form of the energy distribution of electron traps. The energy-resolved distribution of electron traps (ERDT) pattern (Figure S3c) was derived from the differentiation of RDB-PAS spectra. It could be deduced that the number of electron traps in TiO_2_ increased with Ta doping. Works by previous researchers [19,49] have determined that these electron traps are primarily located at the material’s surface. Therefore, the augmented electron traps located at the structure’s surface might have been beneficial for humidity detection because the electrons available in the trap would increase the sensor’s humidity detection capability [50].

The Hall effect measurement results of the samples (Table 2) revealed that the TAFM sample showed a higher carrier concentration at 9.98 × 10^18^ cm^−3^. UTD and TAFM samples exhibited n-type semiconducting characteristics as observed through the Hall effect measurement results. It was expected that pentavalent dopants, such as Ta, would donate extra electrons, increasing the electron concentration in the film, consistent with previous research [51]. The carrier mobility, μ, of the doped sample was higher at 1.92 × 10^3^ cm^2^/V·s compared to 6.35 × 10^2^ cm^2^/V·s for the undoped sample.

Humidity sensors were fabricated using the prepared samples by depositing Ag metal contacts, and the photo images are depicted in Figure S4. The images showed that the Ag contacts and sensing layers of UTD and TAFM were uniformly deposited on the glass substrates.

**Figure 6:**
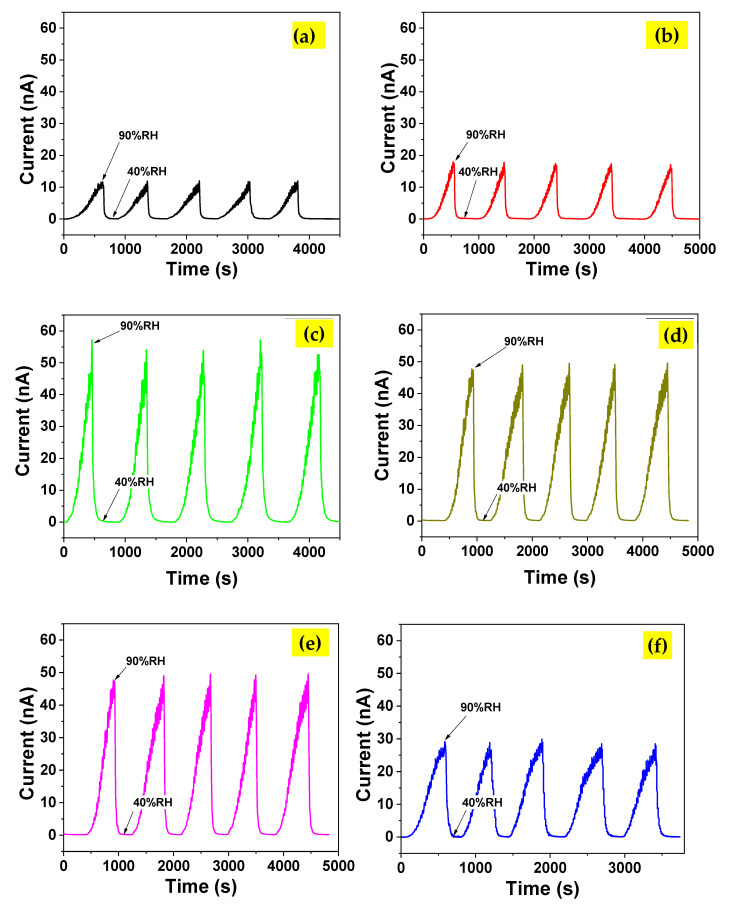
Humidity sensing response of (**a**) UTD, (**b**)TAFM-1, (**c**) TAFM-3, (**d**) TAFM-5, (**e**) TAFM-7, and (**f**) TAFM-9.

The humidity-sensing capabilities of all sensors are shown in Figure 6a. Initially, the humidity inside the chamber was set at 40% RH, and the current value between two metal contacts was measured continuously. The humidity was increased to 90% RH before decreasing to 40% RH to complete one cycle. From the graph, all sensors showed a rapid increase in current as the humidity increased, signalling an excellent response to humidity. The process was repeated over five cycles to determine the sensor response value. The sensor response (*S*) of the sensors was assessed using Equation (5) [52]:

(5) $$S=\frac{R_{40}-R_{90}}{R_{90}}\times 100\%$$

where *R*_40_ is the resistance at 40% RH and *R*_90_ is the resistance at 90% RH.

**Figure 7:**
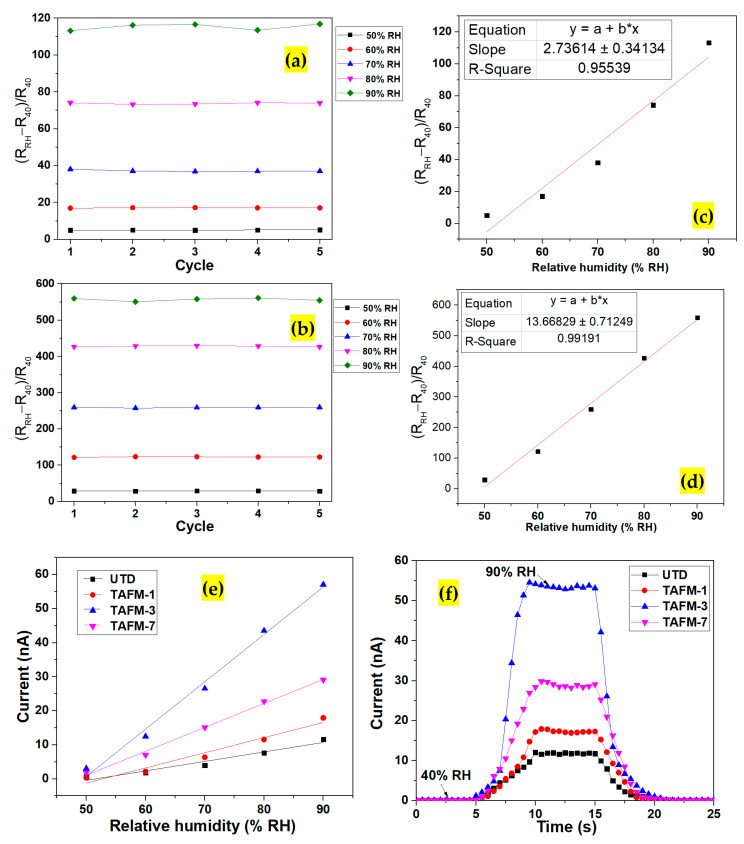
Humidity sensing response of (**a**) UTD and (**b**) TAFM-3 samples over 5 cycles. The transformed humidity sensing response plots of ((R_RH_–R_40_)/R_40_) versus relative humidity for (**c**) UTD and (**d**) TAFM-3. (**e**) Static response of humidity sensors based on UTD, TAFM-1, TAFM-3, and TAFM-7. (**f**) Transient response plots of humidity sensors based on UTD, TAFM-1, TAFM-3, and TAFM-7 to humidity change between 40% RH and 90% RH.

The humidity-sensing responses ((R_RH_–R_40_)/R_40_) of UTD and TAFM-3 samples over five cycles at various relative humidity levels from 50% RH to 90% RH are shown in Figure 7. The plots were constructed by measuring the resistance values of the sensor at specific relative humidity levels for each cycle. The resistance value of the initial humidity level (40% RH) was also measured. The plots (Figure 7a,b) revealed that the sensor’s stability response was exceptionally high for both samples over five cycles with negligible fluctuation. The sensing response versus humidity level plots (Figure 7c,d) for UTD and TAFM-3 produced decent linearity with R-square values of 0.96 and 0.99, respectively. These regression values (R-square) were near 1, indicating that the curves fit to increase linearity.

Meanwhile, the slope of the TAFM-3 sample was higher than that of the UTD sample, indicating that Ta doping improved the responsiveness of humidity sensing. These results also showed that the sensor based on a TAFM-3 sample was highly responsive and stable in the 50–90% RH range.

The static humidity response plots of humidity sensors based on UTD, TAFM-1, TAFM-3, and TAFM-7 at different humidity levels from 40% RH to 90% RH are shown in Figure 7e. The responses of the sensors to changes in humidity level were decent, with the current value rising as the humidity level rose. The plots exhibited good linear characteristics, with slopes of 0.28 ± 0.03 nA/% RH, 0.45 ± 0.05 nA/% RH, 1.39 ± 0.07 nA/% RH, and 0.70 ± 0.03 nA/% RH for UTD, TAFM-1, TAFM-3, and TAFM-7, respectively. The transient response plots of humidity sensors based on UTD, TAFM-1, TAFM-3, and TAFM-7 to humidity change between 40% RH and 90% RH are depicted in Figure 7f. From the transient response plots, the response time and recovery time were calculated to be 5.6 s/4.7 s, 5.1 s/3.7 s, 4.4 s/3.5 s, and 4.9 s/3.8 s, for UTD, TAFM-1, TAFM-3, and TAFM-7, respectively. The response time/recovery time were calculated from the current alteration after the sample was placed in 40 %RH/90% RH condition with a 90% change, for the adsorption and desorption of water molecules, respectively. It is apparent that the Ta doping improved the humidity sensing performance based on these results. The Ta doping contributed to the electronic sensitisation, where it improved the conductivity of the samples by donating electrons. Under low humidity conditions (i.e., 40% RH), the sensing layer’s surface adsorbed oxygen from the atmosphere to form adsorbed oxygen ions (i.e., O^−^ or O_2_^−^) and trapped the surface’s electrons in the process [53]. Those adsorbed oxygen ions formed a depletion layer on the surface of TiO_2_. The Ta-doped samples could attract more oxygen from the environment because of an excess of electrons in the lattice. Upon exposure to the environment that had a higher humidity level (i.e., 90% RH), the adsorbed oxygen species reacted with water molecules on the sensing surface to release the electrons and neutralise the depletion layer. Subsequently, this process restored the electron concentration in the lattice and the film’s conductivity. This process was reversible when the humidity level was reduced to 40% RH. The electron movement was very rapid, which enhanced the response and recovery times of the sensors for the Ta-doped samples compared to their undoped counterparts. The response/recovery times were also affected by the morphology and porosity of the sample, the presence of the active sites on the sample’s surface, the accessibility of hydrophilic functional groups on the sample’s surface, and the ability of the sample to absorb water molecules [54,55].

**Figure 8:**
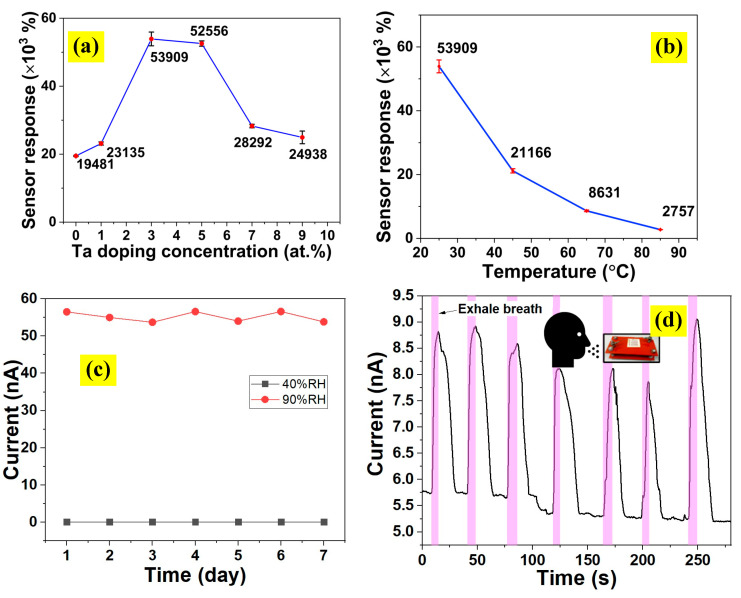
(**a**) Sensory response of TAFM at different Ta concentrations. (**b**) Effect of temperature on the sensor response of the TAFM-3. (**c**) Stability test using the TAFM-3 humidity sensor. (**d**) Sensor response of TAFM-3 toward the human breathing cycle.

The sensor response values for all samples (Figure 8a) showed that the undoped TiO_2_ sensor recorded the lowest response of 11,525%. An improvement was observed as the Ta doping percentage increased, with sample TAFM-3 showing the highest response of 53,909%. The sensing performance was superior or comparable to those reported in other works (Table 3). It was postulated that the higher surface area of TAFM-3, as shown in the FESEM images, contributed to the improved performance of the TAFM-based humidity sensor. The microsphere’s surface was highly porous because of the nanorods’ close arrangement. The humidity detection mechanism in resistive-type sensors, which involves the attachment of water molecules to the TAFM’s surface, benefitted significantly from the highly porous material. As evidenced by the XPS analysis, the increase in oxygen vacancies in the Ta-doped TiO_2_ promoted humidity detection, as reported by Gong et al. [56]. These oxygen vacancy sites dissociated water by transferring one proton to a nearby oxygen atom, increasing the chemisorption of dissociative water molecules. The Ta-doped sample’s response improved because of the increased electron trapping, as proven by the RDB-PAS result. The trapped electrons were released once the water was absorbed into the site, increasing the number of free electrons available for electrical conduction. 

This research also investigated the effects of the surrounding temperature on the sensor performance of the TAFM-3 sample (Figure 8b). The TAFM-3-based sensor response decreased exponentially when the temperature increased from 25 °C to 45 °C, 65 °C, and 85 °C. This phenomenon resulted from the reduced resistance at the base minimum humidity (40% RH) at elevated temperatures. In addition, the sensor at higher temperatures also showed improved signal intensity at maximum humidity (90% RH), reaching up to 79 nA at 85 °C. The increased conductivity at higher temperatures arose from the increased number of free electrons caused by the energy from the surrounding heat. A measurement of the current values at 40% and 90% RH was repeated every day for a week using a TAFM-3 sample to ascertain the stability of the prepared sensor (Figure 8c). Only minimal fluctuation was observed during the measurement period, signalling the device’s high stability. The sensor response toward human breathing was evaluated by measuring the current value change when a human subject breathed near the sensor (Figure 8d). The subject inhaled deeply before exhaling in the sensor’s direction. Sharp increases and decreases in the current were observed, indicating the sensor’s high sensitivity to humidity from human breath. The result indicated that the sensor could detect the presence of human breath.

The proposed humidity detection mechanisms of UTD and TAFM are illustrated in Figure S5. Humidity detection in a resistive-type humidity sensor was achieved by measuring the changes in the electrical resistance of the sensing material upon exposure to water molecules [59]. The large surface area of TAFM microspheres enabled increased adsorption of water molecules. In the initial low-humidity stage, chemisorption and physisorption occur when $H_{2}O$ reacted with –OH through hydrogen bonding, releasing H^+^ ions and allowing protons to hop [60,61]. The ions’ conduction happened through hydronium (H_3_O^+^) ion movement, as shown in Equation (6):

(6) $$H_{2}O+H^{+}\to H_{3}O^{+}$$

A few layers of physiosorbed material formed on the TAFM surface when the humidity increased. The electrostatic force then produced hydronium ions, and the electrical resistance of the sample was governed by ion conduction (Equation (7)):

(7) $$H_{2}O+H_{3}O^{+}↔H_{3}O^{+}+H_{2}O$$

These ions hopped from one hydrogen bond to another in a Grotthuss chain reaction, increasing the sensor’s electrical conductivity [62]. The mechanism shown in Figure S5 shows the current flow of the sensor through the ion conductions during humid conditions under a bias voltage. The depletion layer that existed between the TAFM’s nanorod and its neighbouring nanorod was dependent on the amount of chemisorbed and physiosorbed water molecules [63]. The depletion layer formed when the adsorbed oxygen ions were attached on the surface of the TAFM. At a low humidity level, the depletion layer was thick enough to produce low current output. When more water molecules were adsorbed on the surface of the TAFM at a high humidity level, the thickness of the depletion layer instantly decreased and neutralized, resulting in high current generation. Water molecules condensed in the porous area of the TAFM at a high humidity level also produced conduction bridges between the TAFM’s nanorod and its neighbouring nanorod, enhancing the proton hopping mechanism in the process [63,64,65]. The TAFM and its neighbouring TAFMs also formed interconnected network structures, which allowed current conduction across the film through the easiest path that has the lowest resistance. Comparing UTD and TAFM-3, it was hypothesised that the closely packed arrangement of the TAFM-3 nanorod would produce a higher sensor response than UTD’s branched structure.

**Figure 9:**
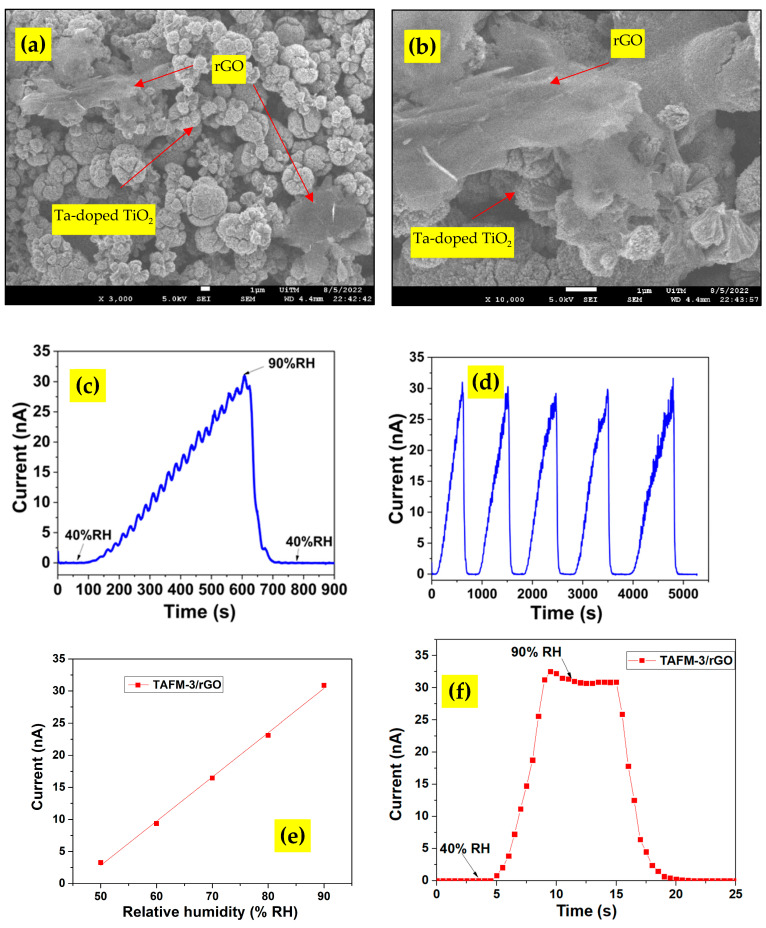
FESEM images of TAFM-3/rGO nanocomposite at (**a**) 3000 times magnification, and (**b**) 10,000 times magnifications, and (**c**) humidity response of Ta-doped TiO_2_/rGO composite. (**d**) Five-cycle reliability measurement of sensor response of Ta-doped TiO_2_/rGO nanocomposite-based humidity sensor. (**e**) Static response of humidity sensor based on TAFM-3/rGO nanocomposite. (**f**) Transient response plot of humidity sensors based on TAFM-3/rGO nanocomposite to humidity change between 40% RH and 90% RH.

A nanocomposite of TAFM-3 and reduced graphene oxide (rGO) was synthesised to explore the possibility of further improvement to the performance of the TFAM-based humidity sensor. The FESEM image of the prepared nanocomposite showed both the TiO_2_ microsphere and rGO sheet structures (Figure 9a,b). The microsphere diameter was equal to the one reported in the previous section. The addition of rGO did not change the dimension of the Ta-doped TiO_2_ microsphere.

The humidity sensor was fabricated using the nanocomposite and the sensor response was observed (Figure 9c). The highest sensor response was 232,152%. The 5-cycle reliability test result (Figure 9d) affirmed the sensor’s reliability with slight fluctuation. Meanwhile, the static humidity response of the nanocomposite in Figure 9e demonstrated excellent linearity, with a slope of 0.69 ± 0.02 nA/% RH. The response and recovery times were 4.2 s and 3.3 s, respectively, as calculated from the transient response plot in Figure 9f. The synergistic and combined effects of doping and nanocomposite contributed to the increased performance of this sensor. When the TiO_2_ microsphere structure increased the adsorption surface area, adding rGO enabled efficient electron transportation between the TiO_2_ microspheres, owing to the excellent electron mobility property of rGO [66]. The rGO acted as a conductive substance to improve the nanocomposite’s conduction. The electrical interaction of TiO_2_ microspheres and rGO subsequently enhanced the humidity response of the film. Moreover, the rGO also contained a large number of active sites and hydrophilic functional groups [26,67], which attracted more water molecules and enhanced the performance of a nanocomposite-based sensor.

## 4. Conclusions

This study synthesised TiO_2_ nanorod-assembled actinomorphic flower-like microsphere structures through Ta doping using a simple solution immersion method and proposed their growth mechanism. The TAFM contained 42-nm nanorod-assembled microspheres with a rutile phase polycrystalline and highly porous structure. The TAFM had higher oxygen vacancy sites, electron trap densities (up to 2.5 μmolg^−1^), electron concentrations (9.98 × 10^18^ cm^−3^), and electron mobilities (1.92 × 10^3^ cm^2^/V·s) as compared to its undoped counterpart. The sensor made from TAFM doped at 3 at.% showed the highest humidity-sensing sensitivity of 53,909%. The formation of a nanocomposite between TAFM-3 and rGO increased the sensor response to 232,152% because of the combined effect of increased surface area and efficient electron transportation.

## Figures and Tables

**Table 1:** Structural properties of the prepared samples analysed from XRD patterns.

Sample	XRD Angle, 2*θ* (º)	Interplanar Distance, *d_hkl_* (Å)	Lattice Parameter, *a* (Å)	Crystallite Size, *D* (nm)	Microstrain, *ε* (×10^−3^)
UTD	26.85	3.32	4.691	47.1	1.017
TAFM-1	26.86	3.31	4.688	40.8	1.166
TAFM-3	26.84	3.32	4.692	25.8	1.857
TAFM-5	26.93	3.31	4.676	23.3	1.980
TAFM-7	27.16	3.28	4.638	23.0	1.852
TAFM-9	26.82	3.32	4.695	19.0	2.543

**Table 2:** Hall Effect measurement result for UTD and TAFM.

Sample	Sheet Resistance (Ω/cm^2^)	Carrier Concentration (cm^−3^)	Carrier Mobility (cm^2^/V·s)
UTD	2.52 × 10^2^	5.00 × 10^18^	6.35 × 10^2^
TAFM-3	3.27 × 10^1^	9.98 × 10^18^	1.92 × 10^3^

**Table 3:** Comparison of humidity sensor performance with other works.

Material	Sensor Type	Humidity Range	Sensor Performance	Ref.
Graphene/ZnO	Resistive	15–86 %RH	S = 7.77 µA/%RH	[24]
Ti_3_C_2_/TiO_2_	Capacitive	7–97 %RH	S = 1614 pF/%RH	[57]
Titanium Carbide	Resistive	33–95 %RH	$S=15\%\;[\left(\Delta R/R_{o}\right)\times 100\%]$	[52]
TiO_2_	Resistive	5–95 %RH	S = 4940%	[58]
Ta-doped TiO_2_	Resistive	40–90 %RH	$S=53,909\%\;[\left(\Delta R/R_{o}\right)\times 100\%]$	Our work

## Data Availability

Data is contained within the article.

## Supplementary Materials

The following supporting information can be downloaded at:https://www.mdpi.com/article/10.3390/nano13020256/s1, Figure S1: Sample preparation setup for TiO_2_ solution immersion method; Figure S2: FESEM image of: (a) UTD and (b) TAFM-3 at 30,000× magnification; Figure S3: RDB-PAS measurement results of: (i,ii,iii) UTD and (iv,v,vi) TAFM-3; Figure S4: Photo image of humidity sensor made of: (a) UTD, (b) TAFM-1, (c) TAFM-3, (d) TAFM-5, (e) TAFM-7, and (f) TAFM-9; Figure S5: (a) Humidity detection mechanism of UTD and TAFM-3. (b) Current flow of humidity sensor induced by ionic condition under humid condition. (c) Proton hopping mechanism between the nanorod in the TAFM and the nanorod in the adjacent TAFM.
