# Supplementary material for: Formation of a Nanorod-Assembled TiO2 Actinomorphic-Flower-like Microsphere Film via Ta Doping Using a Facile Solution Immersion Method for Humidity Sensing
Source: Nanomaterials (Basel). 2023 Jan 6;13(2):256. doi: 10.3390/nano13020256 (PMC9861450; doi:10.3390/nano13020256)
Supplement: Supplementary file 1 [file nanomaterials-13-00256-s001.zip › nanomaterials-2098865-supplementary materials.pdf]

Supplementary Materials

Formation of a Nanorod-Assembled TiO_2 Actinomorphic-Flower-like Microsphere Film via Ta Doping Using a Facile Solution Immersion Method for Humidity Sensing

Musa Mohamed Zahidi [1,2], Mohamad Hafiz Mamat [1,*], A Shamsul Rahimi A Subki [1,3], Mohd Hanapiah Abdullah [1,2], Hamizura Hassan [4], Mohd Khairul Ahmad [5], Suriani Abu Bakar [6], Azmi Mohamed [6] and Bunsho Ohtani [7]

[1] NANO-ElecTronic Centre (NET), School of Electrical Engineering, College of Engineering, Universiti Teknologi MARA, Shah Alam 40450, Selangor, Malaysia;
[2] Centre for Electrical Engineering Studies, Universiti Teknologi MARA Cawangan Pulau Pinang, Permatang Pauh 13500, Pulau Pinang, Malaysia
[3] Faculty of Electrical and Electronic Engineering Technology, Universiti Teknikal Malaysia Melaka, Hang Tuah Jaya, Durian Tunggal 76100, Melaka, Malaysia
[4] Centre for Chemical Engineering Studies, Universiti Teknologi MARA Cawangan Pulau Pinang, Permatang Pauh 13500, Pulau Pinang,
[5] Microelectronic and Nanotechnology—Shamsuddin Research Centre, Faculty of Electrical and Electronic Engineering, Universiti Tun Hussein Onn Malaysia, Batu Pahat 86400, Johor, Malaysia;
[6] Nanotechnology Research Centre, Faculty of Science and Mathematics, Universiti Pendidikan Sultan Idris, Tanjung Malim 35900, Perak, Malaysia;
[7] Graduate School of Environmental Science, Hokkaido University, Sapporo 060-0810, Japan;
* Correspondence: mhmamat@uitm.edu.my; Tel.: +603-552-1182

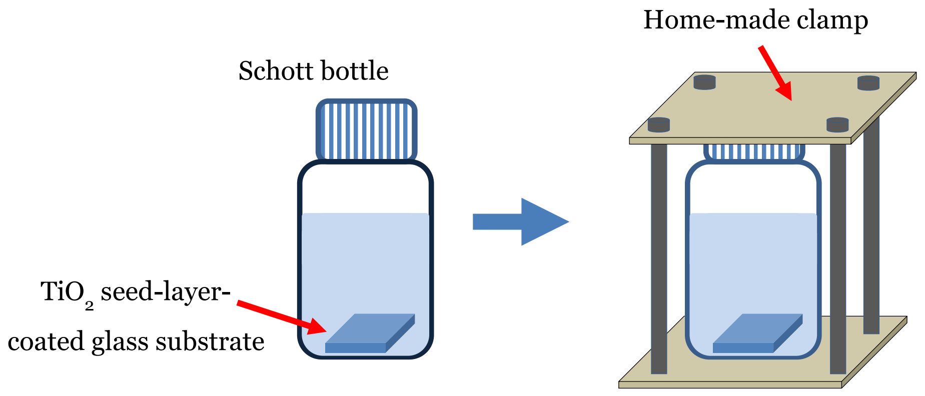

Figure S1. Sample preparation setup for TiO_2 solution immersion method.

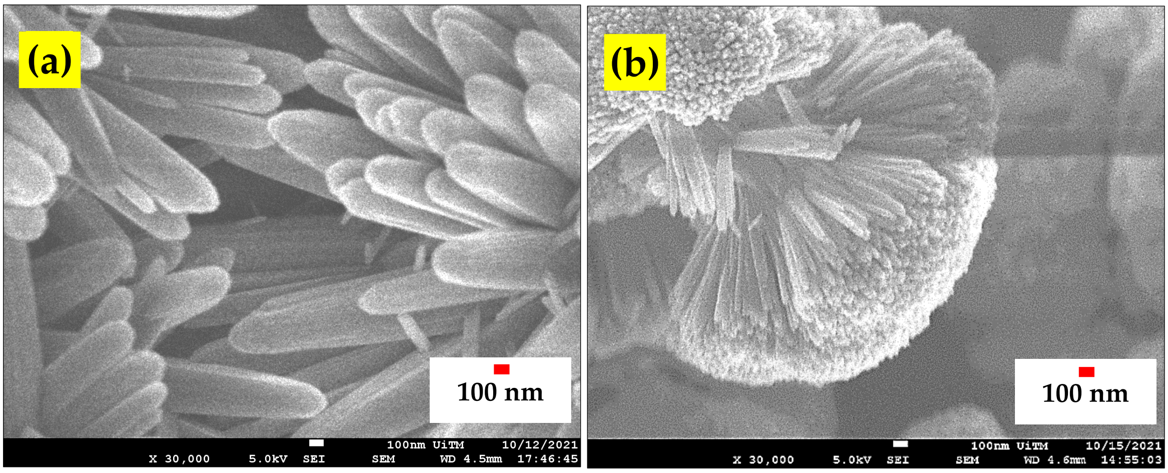

Figure S2. FESEM image of: (**a**) UTD and (**b**) TAFM-3 at 30,000 × magnification.

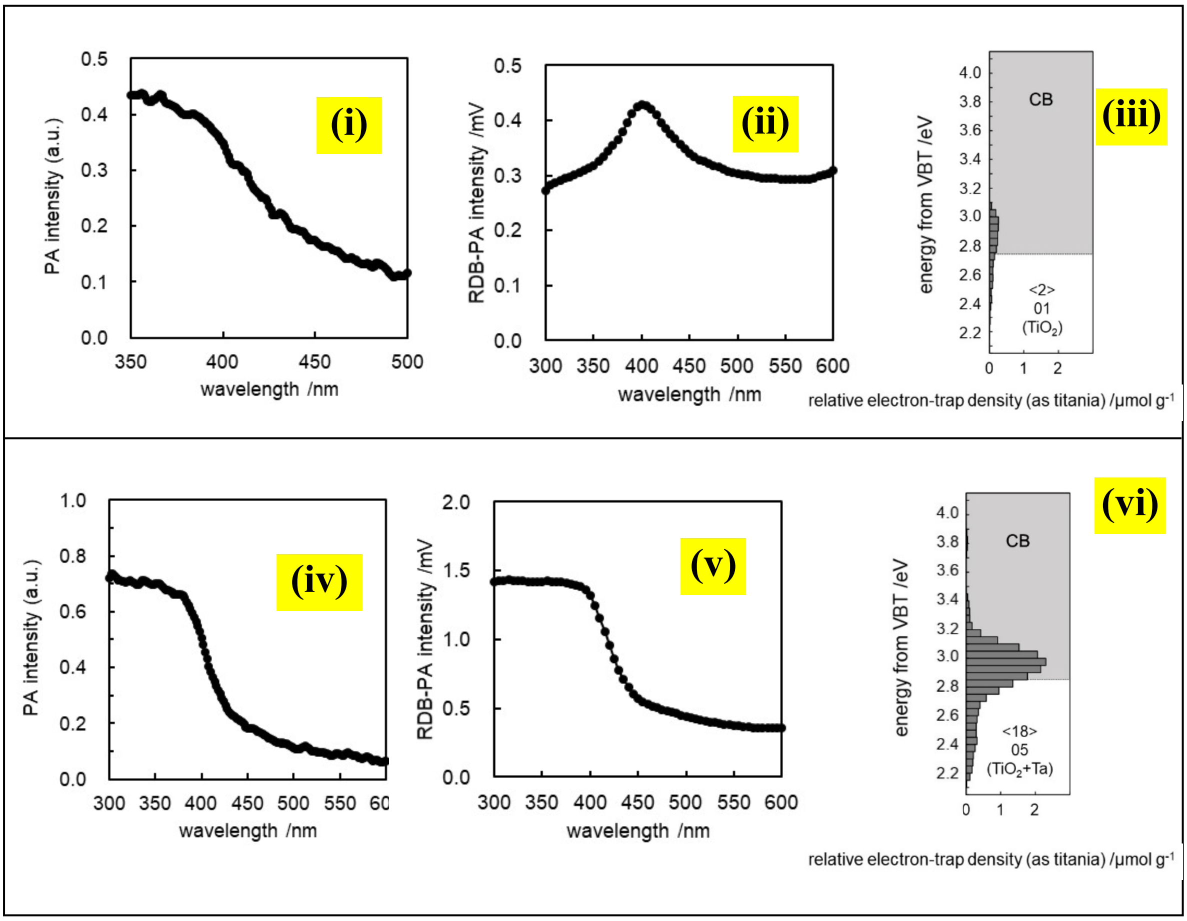

Figure S3. RDB-PAS measurement results of: (**i–iii**) UTD and (**iv–vi**) TAFM-3.

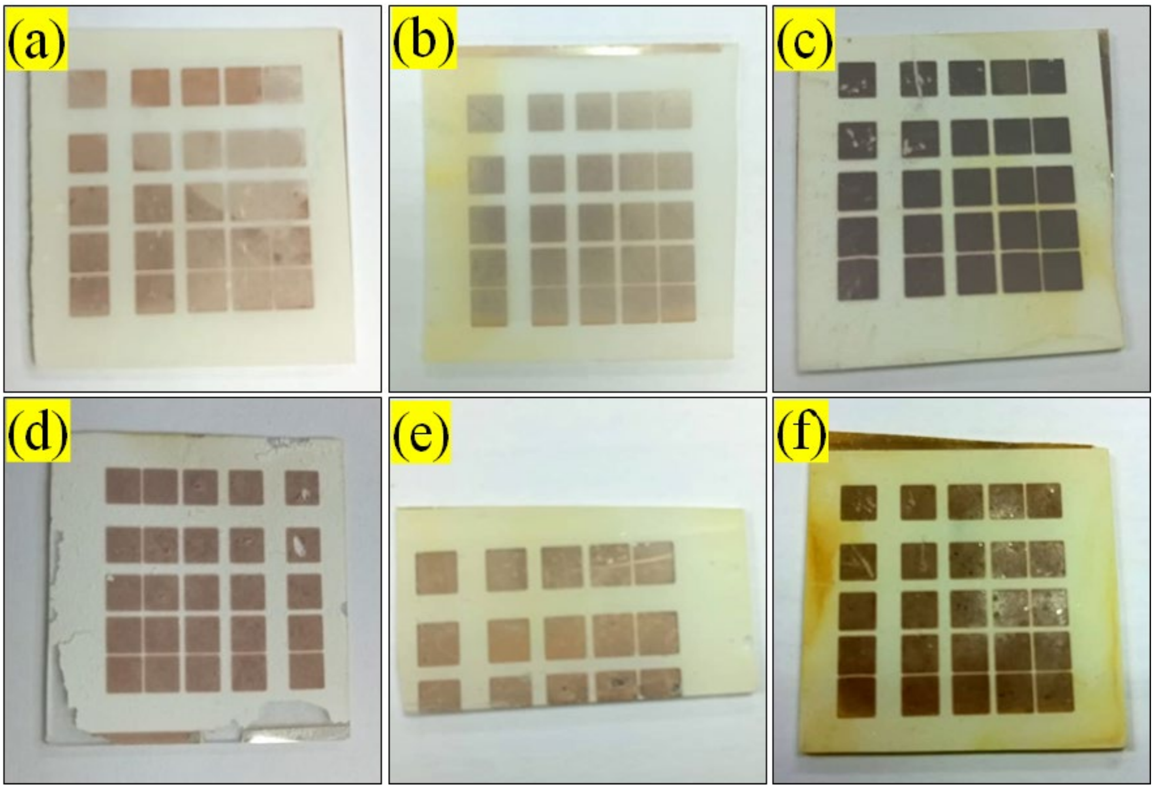

Figure S4. Photo image of humidity sensor made of: (**a**) UTD, (**b**) TAFM-1, (**c**) TAFM-3, (**d**) TAFM-5, (**e**) TAFM-7, and (**f**) TAFM-9.

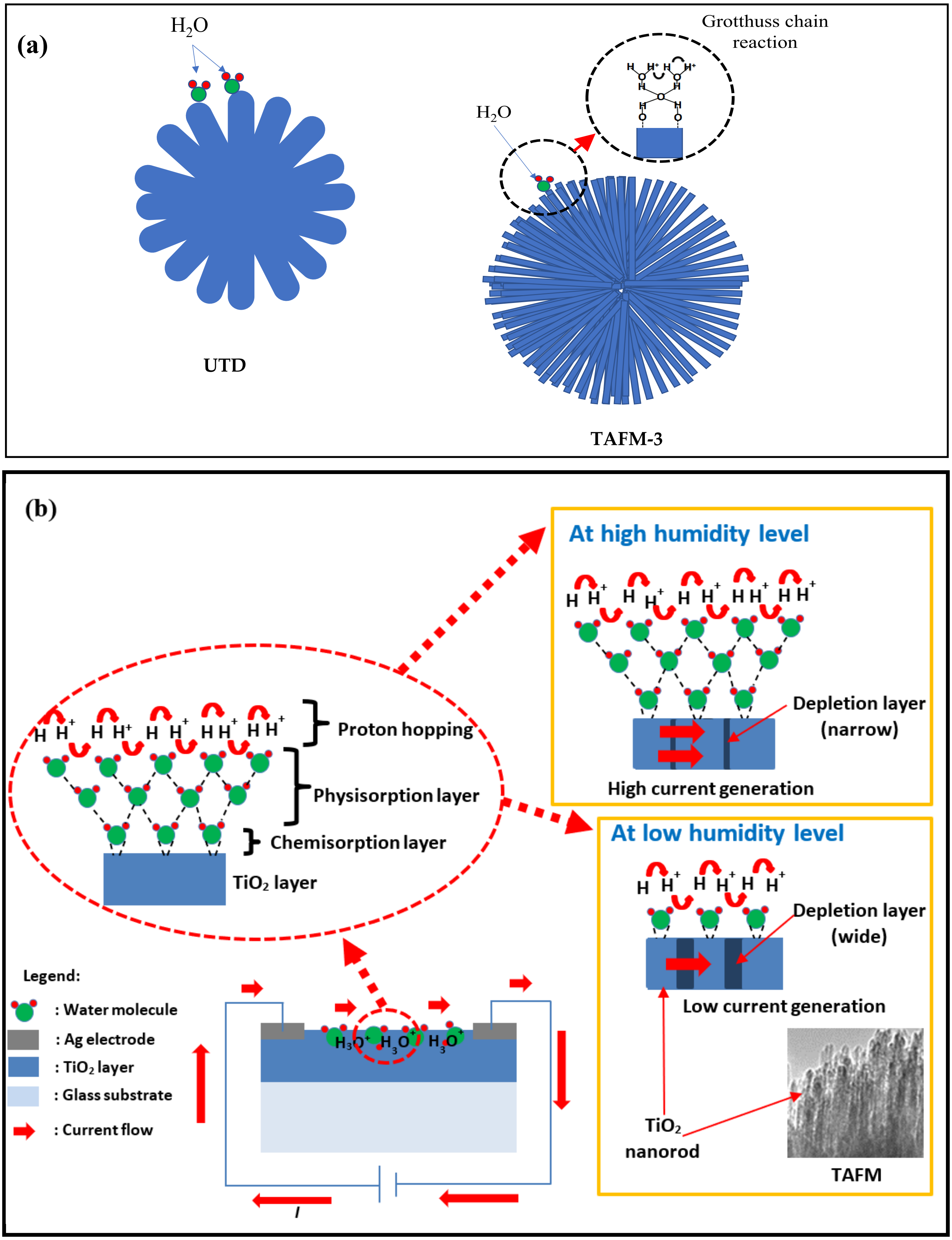
(a)
H2O
UTD
Grotthuss chain reaction
H2O
TAFM-3
(b)
At high humidity level
Depletion layer (narrow)
High current generation
At low humidity level
Depletion layer (wide)
Low current generation
Proton hopping
Physisorption layer
Chemisorption layer
TiO2 layer
Legend:
: Water molecule
: Ag electrode
: TiO2 layer
: Glass substrate
: Current flow
H3O+ H3O+ H3O+
I
TiO2 nanorod
TAFM

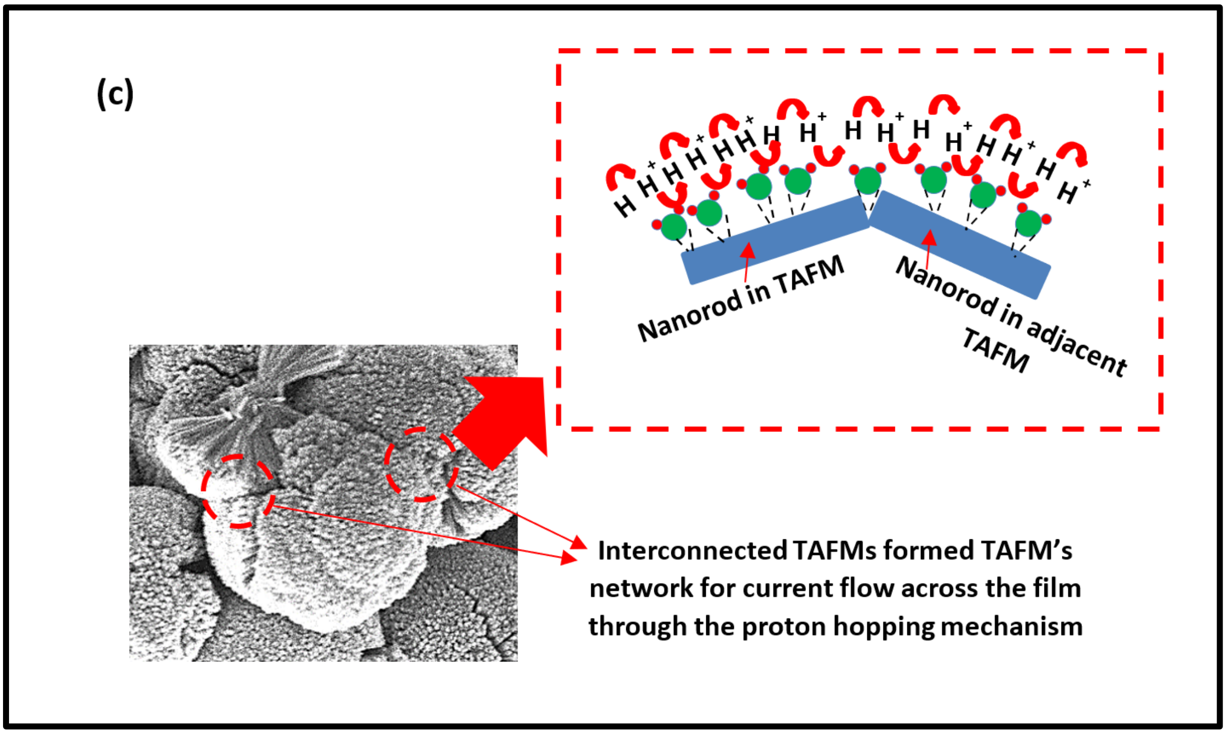

Figure S5. (**a**) Humidity detection mechanism of UTD and TAFM-3. (**b**) Current flow of humidity sensor induced by ionic conduction under humid condition. (**c**) Proton hopping mechanism between the nanorod in the TAFM and the nanorod in the adjacent TAFM.
